# Molecular and Immunohistochemical Markers with Prognostic and Predictive Significance in Liver Metastases from Colorectal Carcinoma

**DOI:** 10.3390/ijms19103014

**Published:** 2018-10-03

**Authors:** Gianluca Lopez, Francesca Boggio, Stefano Ferrero, Nicola Fusco, Alessandro Del Gobbo

**Affiliations:** 1 Division of Pathology, Fondazione IRCCS Ca’ Granda Ospedale Maggiore Policlinico, via Francesco Sforza 35, 20122 Milan, Italy; gianluca.lopez@outlook.com (G.L.); francesca.boggio@hotmail.it (F.B.); stefano.ferrero@unimi.it (S.F.); 2 Department of Biomedical, Surgical and Dental Sciences, University of Milan, via Francesco Sforza 35, 20122 Milan, Italy

**Keywords:** biomarkers, immunohistochemistry, molecular markers, colorectal cancer, liver metastases, prognostic, predictive

## Abstract

Despite the significant recent achievements in the diagnosis and treatment of colorectal cancer (CRC), the prognosis of these patients has currently plateaued. During the past few years, the opportunity to consider multiple treatment modalities (including surgery and other locoregional treatments, systemic therapy, and targeted therapy) led to the research of novel prognostic and predictive biomarkers in CRC liver metastases (CRCLM) patients. In this review, we seek to describe the current state of knowledge of CRCLM biomarkers and to outline impending clinical perspectives, in particular focusing on the cutting-edge tools available for their characterization.

## 1. Introduction

Colorectal cancer (CRC) is the third most common cancer in men and the second in women across all regions of the world, with approximately 1.3 million newly diagnosed cases every year [1,2,3]. This neoplasm represents the fourth most common cause of cancer-related death worldwide [1]. To date, more than 90% of early-stage CRC patients are alive after five years from diagnosis [1,4]. However, in the case of regional spread to adjacent organs or lymph nodes, the 5-year relative survival rate decreases to 69%, and when distant metastases are present, it dramatically drops to approximately 10% [4]. Despite the significant recent achievements in the diagnosis and treatment of these patients, the prognosis of CRC has currently plateaued. This could be due, at least in part, to the lack of a complete understanding of the biology which underpins the metastatic process of CRC.

Given its extremely abundant blood supply from the colon and rectum, the liver is the dominant metastatic site for patients with CRC [5]. Hence, more than 50% of patients will develop liver metastases within three years after diagnosis, and although the majority of affected patients have multi-organ dissemination, in one-third of cases the disease is isolated to the liver [6,7]. In these patients, resection of the liver metastases offers the best chance of long-term survival as an alternative to or in combination with systemic chemotherapy [7]. Regrettably, even when resection is combined with adjuvant systemic regimens, it is curative in only 20% of CRC liver metastasis (CRCLM) [8,9]. Furthermore, the number of patients eligible for liver resection, albeit increased with the introduction of portal vein embolization techniques, remains relatively low [8].

During the past few years, the opportunity to consider multiple treatment modalities (including surgery and other locoregional treatments, systemic therapy, and targeted therapy) lead to the research of novel prognostic and predictive biomarkers in CRCLM patients. In this review, we seek to describe the current state of knowledge of CRCLM biomarkers and to outline impending clinical perspectives, in particular focusing on the cutting-edge tools available for their characterization.

## 2. Results

### 2.1. Molecular Landscape and Clonal Evolution Phenomena in CRCLM

**Figure 1:**
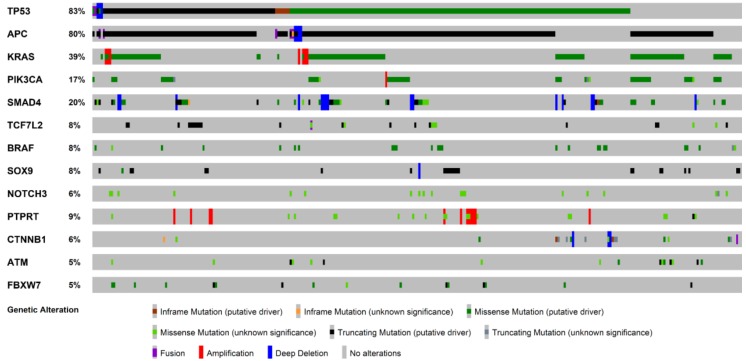
Oncoprint visualization of highly recurrent somatic molecular alterations by frequency in colorectal cancer liver metastases (313 patients, 319 samples from cBioPortal). Each row represents a gene, as reported on the left; types of alterations are color-coded on the basis of the legend on the bottom.

**Figure 2:**
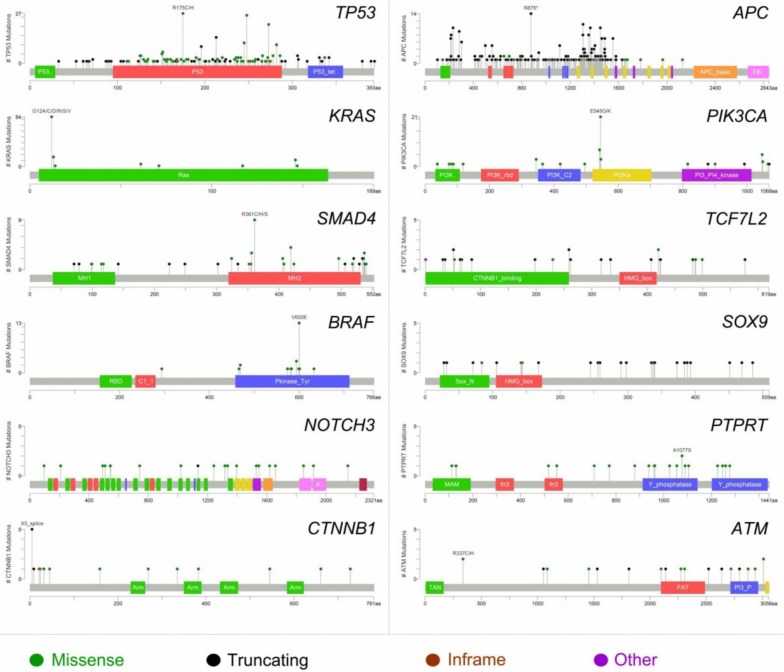
Domain structure and gene alterations of the 12 most frequently altered genes in colorectal cancer liver metastases (312 patients, 318 samples from cBioPortal). Mutation types are color-coded on the basis of the legend at the bottom.

**Figure 3:**
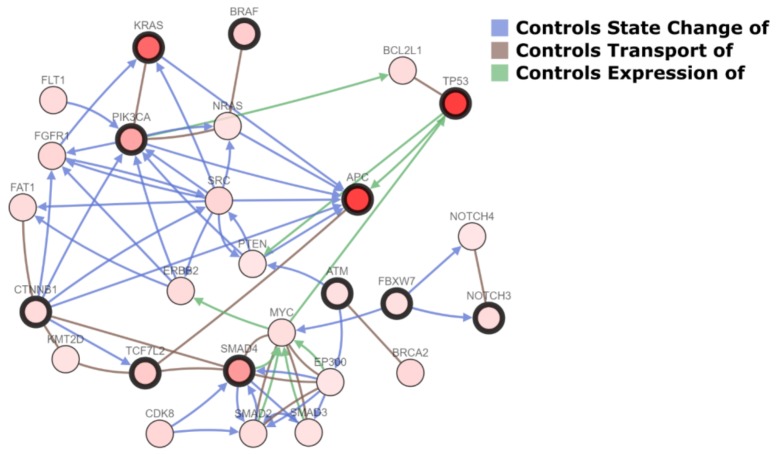
Network of the interactions between the most frequently altered genes in colorectal cancer liver metastases (highlighted in bold) and other cancer genes. Interaction types (arrows and lines) are color-coded on the basis of the legend at the top right.

Metastatic CRC has been shown a remarkable degree of tumor-to-metastasis heterogeneity, with a number of altered genes which are specific to the primary tumor and others of the metastasis [10]. Numerous lines of evidence demonstrate that this heterogeneity, not only for CRC, is due to phenomena of clonal selection that follows a Darwinian evolutionary model and has crucial clinical implications [10,11,12,13,14,15,16,17]. Hence, investigation of the mutation status only in the primary tumor has limitations as an indicator for the selection of treatment of CRCLM. Several tumor suppression genes (e.g., *TP53* and *CDKN1A*), Ki67 proliferation index, and markers of angiogenesis (e.g., microvessel density and thrombospondin-1) have been found to predict tumor recurrence and survival, as confirmed by the preliminary data of ongoing clinical trials [18,19,20,21,22,23,24,25,26,27,28]. Furthermore, massively parallel sequencing studies have recently shown that shared mutations between CRC and the corresponding CRCLM are present in 50 to 96% of cases, while approximately 10% of cases show the presence of private somatic mutations in the liver metastases [10]. As also demonstrated by the analysis of publicly available genomic data [29], the most recurrently mutated genes in CRCLM are *TP53*, *APC*, *KRAS*, *PIK3CA*, *SMAD4*, *TCF7L2*, *BRAF*, *SOX9*, *NOTCH3*, *PTPRT*, *CTNNB1*, *ATM*, and *FBXW7* (Figure 1). Most of the molecular scars targeting these genes are missense mutations, while truncating (putative driver) mutations show high frequencies in *TP53* and *APC* [29]. Characteristically, the recurrently mutated genes in CRC, with the exception of *PIK3CA* and *SMAD4*, show a low degree of discordance between the primary tumor and the synchronous liver metastasis, confirming their biological role as driver “trunk” mutations [10]. Furthermore, most of the genetic alterations in CRCLM are hotspot mutations, in accordance to the data provided by The Cancer Genome Atlas Network (TCGA), as shown in Figure 2. With the exception of *BRAF*, multiple passenger mutations are frequently observed in CRCLM. These molecular alterations do not have a well-defined clinical role, confirming the complexity of CRC molecular landscape. It is of note that only a few mutations are recurrent in metastatic CRC patients, indicating that this neoplasm represents a heterogeneous constellation of different diseases rather that a single condition [10,19]. Hence, most of the recurrently mutated genes in metastatic CRC are biologically interconnected, as represented in Figure 3. These data add further evidence to the body of literature suggesting that individualized treatment strategies are warranted also in metastatic CRC patients showing highly recurrent mutations [19,20,30,31].

### 2.2. The Prognostic Role of SMAD4, KRAS, and BRAF.

**Figure 4:**
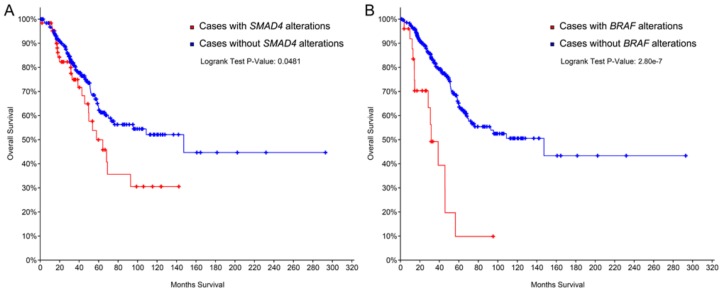
Overall survival of 312 colorectal cancer patients with liver metastases based on *SMAD4* (**A**) and *BRAF* (**B**) gene alterations. Survival curves are built according to the Kaplan–Meier method. Data from The Cancer Genome Atlas Network are publicly available at cbioportal.org.

Recent studies have provided evidence on the prognostic and likely predictive role of *SMAD4* and *BRAF*, two genes that are currently considered tumor suppressors in CRC. In particular, the overexpression in CRCLM of *SMAD4* leads to a worse prognosis following liver resection [18]. Furthermore, the loss of *SMAD4* expression and elevated Ki67 expression significantly correlate with liver metastasis, regardless of the time of occurrence [32]. Analysis of publicly available genomic data further corroborates this concept (Figure 4A). Taken together, both the overexpression and presence of gene somatic mutations of *SMAD4* in liver disease are bona fide indicators of a more aggressive disease. On the other hand, overexpression of *SMAD4* in liver parenchyma has been proposed as a predictor of chemotherapy response [32,33].

Several research groups have provided evidence that two proto-oncogenes, *KRAS* and *BRAF*, are associated with resistance to chemotherapy and poor prognosis [34,35,36,37,38,39,40,41,42,43], as confirmed by the survival analysis of the *BRAF* mutated CRCLM included in the TCGA cohort (Figure 4B).

### 2.3. miRNA Expression Profiling

MicroRNAs (miRNAs) are a noncoding part of the genome consisting in small ribonucleic acid sequences (less than 25 nucleotides) controlling cellular and physiological processes by interfering with the translation of messenger RNA (mRNA) to proteins [44,45]. They are involved in cancer development, progression, and metastasis [46,47,48]. Affymetrix microarrays involving 1036 miRNAs were performed in a large study including two pairs of primary CRCs and their matched liver metastases; results were validated using quantitative real-time polymerase chain reaction assay. In particular, 13 miRNAs were deregulated in primary CRCs compared to their matched liver metastases, with a significantly reduced expression of miR-99b-5p, miR-377, and miR-200c, and increased expression of miR-196b-5p in the liver metastasis [47,49,50]. In addition, miR-200c and miR-196b-5p were positively correlated with shorter overall survival in patients with liver metastasis [51]. Another study of gene expression arrays and microRNA profiling showed higher expression of c-met and concomitant reduction of miR-146a in the metastatic variants in murine MC38 adenocarcinoma cells [47]. Expression levels of both c-met and miR-146a were similar between primary tumors and liver metastases. In addition, overexpression of miR-146a in metastatic clones showed reduced in vitro malignancy and abolished the development of primary tumor and liver metastases. Correlation between miR-181a expression between patients with and without liver metastasis using qRT-PCR showed how higher levels of this marker are associated with advanced stage and development of distant metastasis, identifying miR-181a as an independent prognostic factor of poor overall survival [52]. miR-9 has been reported to be involved in the metastasis of several malignancies including brain breast cancer, and in colorectal cancer has been demonstrated to be involved in the metastatic process facilitating cell motility through downregulation of α-catenin expression in RKO cells, without interfering with cell proliferation [53]. miR-21 has been identified to be upregulated in exosomes, primary tumor tissues, and liver metastasis tissues [47,54,55,56]. In detail, exosomal miR-21 showed a significant association with liver metastasis and TNM stage, and overall and disease-free survival in high-exosomal-miR-21 patients were significantly worse than those in low-miR-21 patients with TNM stage II or III, and for overall survival-only in patients with TNM stage IV (i.e., metastatic disease) [54].

### 2.4. EMX2

Empty Spiracles Homeobox 2 (*EMX2*) gene has been associated with neuronal growth and differentiation [57]. qtRT-PCR technology and siRNA-mediated knockdown and adenoviral delivery-mediated overexpression of EMX2 were performed showing that colorectal tumor samples had decreased EMX2 expression levels, and its downregulation was associated with distant metastasis concluding that overexpression of EMX2 led to an inhibition of tumor cell migration [57].

### 2.5. DYRK2

Dual-specificity tyrosine-regulated kinase 2 (*DYRK2*) controls the epithelial-to-mesenchymal transition in breast cancer and ovarian serous adenocarcinoma [58]. An in vivo xenograft model showed that the ectopic expression of DYRK2 correlated with diminished metastatic lesion since cell invasion and migration was abolished by overexpression of DYRK2. Lower expression of DYRK2 levels in liver metastases correlated with worse overall and disease-free survival, allowing for the consideration of DYRK2 as a predictive marker for liver metastases of colorectal cancer [58].

### 2.6. Chromosome 20p11 Gains

Screening for chromosomal aberrations by microarray-based comparative genomic hybridization showed that twelve genes mapping at 20p11 were significantly overexpressed as a consequence of 20p11 copy number gain [59]. This correlated with significantly higher C20orf3 protein expression in patients with hepatic metastases than in those with extrahepatic metastases [59].

### 2.7. GADD45B

Growth arrest and DNA-damage-inducible beta (*GADD45B*) is a member of the growth arrest DNA damage-inducible gene family and plays a crucial role in DNA damage repair, cell growth, and apoptosis. The relationship between GADD45B expression and colorectal cancer progression has been recently investigated, and the results showed that overexpression of GADD45B is associated with poorer prognosis for CRC patients both in overall survival and disease-free survival and that, regarding protein levels, GADD45B was gradually upregulated in normal mucosa, primary tumors, and liver metastases tissues in ascending order, with a prognostic role in terms of progression. The authors underlined the significant longer overall survival and disease-free survival among patients with low GADD45B expression pointing at the high expressor-subgroup as one which might benefit from adjuvant chemotherapy [60].

### 2.8. CD133^+^ CD54^+^ CD44^+^ Circulating Tumor Cells

The expression of the CD133^+^ CD54^+^ CD44^+^ cellular subpopulation of circulating tumor cells has been identified as significantly associated with liver metastasis of colorectal cancer [61,62,63,64]. In a recent study, flow cytometry showed that the liver metastatic CRC patients with high expression of CD133^+^ CD54^+^, CD133^−^ CD54^+^, and CD133^+^ CD44^+^ CD54^+^ cellular subpopulations of circulating tumor cells had a worse survival than those patients with low expression. Moreover, the cellular subpopulation expressing CD133^+^ CD44^+^ CD54^+^ of CTCs showed its proficiency as independent prognostic factors for survival in CRC patients with liver metastasis not treated with surgical approach [65].

### 2.9. TGF-Beta

Transforming growth factor-beta (TGF-β) plays a role in the development of colorectal cancer from normal mucosa and in its metastatic process [66,67]. The CRC subtype with a mesenchymal and aggressive phenotype having TGF-β as a hub gene of this signature has been investigated in recent genomic studies. The inhibition of TGF-β signaling has been shown to impair experimental CRC metastasis to the liver [66].

### 2.10. TBL1XR1

The role of transducin (β)-like 1 X-linked receptor 1 (TBL1XR1) in predicting liver metastasis for early stage CRC has been evaluated in a large study with both immunohistochemical and RT-qPCR techniques. For stage IV CRC patients, TBL1XR1 expression correlated with the number of liver metastases and high levels of TBL1XR1 in liver metastases indicated poor overall survival [68]. In addition, high expressions of TBL1XR1 predicted for liver metastasis in early-stage CRC patients [69].

### 2.11. SDF1

Stromal-derived cell factor-1 (SDF1), also known as CXCL12, is a chemokine involved in developmental processes and maintenance of tissue stem cells [70]. A study by Amara et al. [71] showed that the high expression of SDF1 in colorectal cancer and in the associated liver metastases was correlated with higher tumor grade, advanced clinical stage, and lymphatic invasion; therefore, SDF1 seems to have an indirect prognostic significance. This study also suggests that this protein may play a role in the promotion of the metastatic process.

### 2.12. Galectin-3

Galectin-3 is a protein involved in cell proliferation, adhesion, differentiation, angiogenesis, and apoptosis in normal and pathologic tissues; recent studies indicate that Galectin-3 plays a role in tumor cell transformation and metastasis [72]. Arfaoui-Toumi et al. [73] observed a progressive decrease of galectin-3 in primary tumor tissue and in liver metastases, correlating with lower degrees of tumoral differentiation in non-mucinous carcinomas.

### 2.13. FRalpha

Folate receptor alpha (FRalpha) is a membrane transport protein with high affinity for folic acid; FRalpha levels are higher in specific malignant tumors of epithelial origin compared to normal tissues with a positive association to tumor stage and grade [74]. A study by D’Angelica et al. [75] demonstrated that FRalpha positivity in resectable hepatic colorectal metastases was associated with the early death group of patients (<2-year survival), independently of clinical risk score and margin.

### 2.14. Ki67

Ki67 is a protein without a clearly defined function; it has been associated with ribosomal RNA synthesis, and is expressed during cell proliferation; the fraction of cells that express this protein, assessed by immunohistochemistry, represent the proliferative subset of a cellular population [76]. A study by Eefsen et al. [77] showed that in liver metastasis from colorectal cancer, a high proliferation index assessed by Ki67 expression correlated to a shorter recurrence-free survival. In contrast, a study by D’Angelica et al. [75] observed no significant difference in terms of Ki67 expression between the early death group (<2-year survival) and the long survival group (>10-year survival).

### 2.15. MMP7

Matrix metalloproteinase-7 (MMP7) is a member of matrix metalloproteinases, a family of endopeptidases involved in degradation of extracellular matrix, which leads to tissue repair and remodeling, and is implicated in various physiological as well as pathological processes [78]. A study by Fang et al. [79] showed that expression of this protein in the primary tumor tissue could be used as a predictor tool of liver metastases.

### 2.16. CCL15

Chemokine (C–C motif) ligand 15 (CCL15) is a small cytokine belonging to the CC chemokine family. A study by Itatani et al. [80] demonstrated that liver metastases with an expression of CCL15 contain a high number of CCR1^+^ myeloid cells that produce matrix metalloproteinase 9 and are associated with shorter disease-free survival by promoting tumor invasion and metastasis.

### 2.17. pIgR

Polymeric immunoglobulin receptor (pIgR) is a transmembrane protein involved in the immunologic response [81]. A study by Liu et al. [82] observed that patients with positive pIgR expression in colon carcinoma hepatic metastasis had a significantly worse prognosis than patients with negative expression of this protein.

### 2.18. Endoglin

Endoglin (CD105) is a cell membrane receptor for transforming growth factor β and bone morphogenetic proteins involved in angiogenesis [83]. A study by Mitselou et al. [84] showed that high expressions of endoglin in the primary tumor site are associated with liver metastasis.

### 2.19. Nek2

Never in Mitosis Related Kinase 2 (Nek2) is a protein involved in regulating mitotic processes, including centrosome duplication and separation, microtubule stabilization, kinetochore attachment, and spindle assembly checkpoint [85]. A study by Neal et al. [86] proved that Nek2 overexpression in colorectal cancer primary tumor site and in its liver metastases is associated with a shortened cancer-specific survival.

### 2.20. Beta-Catenin

Beta-catenin is a protein involved in the regulation and coordination of cell-cell adhesion and gene transcription [87]. A study by Pancione et al. [88] and demonstrated that reduction or loss of beta-catenin expression in the primary tumor was associated with liver metastases. On the other hand, another study by Wang et al. [89] showed that primary tumor nuclear overexpression of beta-catenin correlates with liver metastases.

### 2.21. PPARG

Peroxisome proliferator-activated receptor gamma (PPARG) is a nuclear receptor involved in glucose and fatty acids metabolism [90]. A study by Pancione et al. [88] showed that reduction or loss of PPARG expression in the primary tumor was associated with liver metastases.

### 2.22. MACC1

MACC1 is a key regulator of the hepatocyte growth factor (HGF) receptor (HGFR or MET) pathway, which is involved in cellular growth, epithelial–mesenchymal transition, angiogenesis, cell motility, invasiveness, and metastasis. A study by Ren et al. [91] observed that high expression of MACC1 in the primary tumor site is associated with liver metastasis.

### 2.23. MET

MET is a tyrosine kinase receptor essential for embryonic development, organogenesis, and wound healing [92]. A study by Ren et al. [91] observed that high expression of MET in the primary tumor site is associated with liver metastasis.

### 2.24. CA9

Carbonic anhydrase 9 (CA9) is a member of the carbonic anhydrases family, a class of zinc metalloenzymes involved in a variety of biological processes. A study by Van den Eynden et al. [93] demonstrated that CA9 correlates with liver metastases angiogenesis, which in turn is correlated with the “pushing” growth pattern of the metastatic lesion; this particular histologic type of liver metastasis has been shown in the same study to have a poor prognosis.

### 2.25. Beta-1 Integrin

Beta-1 integrin is a member of a family of proteins involved in cell adhesion and recognition in a variety of processes including embryogenesis, hemostasis, tissue repair, immune response, and metastatic diffusion of tumor cells [94]. A study by Vassos et al. [95] observed that beta-1 integrin expression correlates with the histological grade of colorectal cancer liver metastases; therefore, this marker has indirect prognostic significance and can aid in the evaluation of metastases grading.

### 2.26. MLH1/PMS2

MLH1 and PMS2 are part of the DNA mismatch repair (MMR) system, a cellular process involving different proteins, resulting in the identification and subsequent repair of mismatched bases [96]. A study by Larsen et al. [97] demonstrated that in colorectal cancers, deficiency of MLH1/PMS2 in the primary tumor tissue is correlated with an MLH1/PMS2 proficiency in the associated liver metastases. This phenomenon could have implications for the metastases chemosensitivity.

### 2.27. Tenascin C

Tenascin C has been identified as a predicted target of miR-198, one of the top 10 miRNAs downregulated in tumor stroma of CRCs with synchronous liver metastasis.

Consequently, tenascin C protein expression in primary CRCs revealed that a high staining intensity was correlated with synchronous liver metastasis and its staining intensity was an independent prognostic factor to predict postoperative overall survival and liver metastasis-free survival [98].

## 3. Discussion

In this review, we identified the most recent studies involving molecular and immunohistochemical markers associated with a more aggressive phenotype in CRCML, and those that can give prognostic and/or predictive information in stage IV patients. In contrast to the relative abundance of published research in literature regarding the aforementioned categories of biomarkers in primary CRC (an input of the search terms “prognostic predictive biomarkers colorectal carcinoma” in the search engine PubMed restricted to the last 10 years produced 813 results), there is a relative shortage of studies focused on this particular topic in the liver metastases of this malignancy (as discussed in the Methods section, primary screening identified 394 studies, of which only 110 were relevant to this review). The prognostic stratification of patients with a metastatic disease is however crucial, given their poor 5-year overall survival rate (11.7%) [8]; in this setting, novel biomarkers with prognostic and predictive value are urgently needed, given the fact that the sole morphological evaluation of hematoxylin and eosin slides of resections of liver metastases, apart from giving invaluable information about the surgical margin (indicating the radicality of the surgical operation), does not give any more prognostic information, and has no predictive value in terms of response to target therapies.

A subset of molecular biology techniques (i.e., RT-PCR, NGS, and blotting techniques) allows, to date, to give both prognostic and predictive information in a variety of human malignancies. In addition, immunohistochemical techniques, i.e., the identification of antigen expression in the nucleus, cytoplasm or cellular membrane in tumoral cells through an antigen–monoclonal antibody reaction, are widely used in routinary histopathological practice, and allow the identification of markers that can be associated with a peculiar tumoral phenotype or different prognosis in a number of human malignancies such as CRC. However, the value of such techniques in particular settings such as metastatic diseases eligible for surgical resection (such as CRCLM), is not always well-defined as in their primary counterpart.

In this view, apart from the urgent need of novel biomarkers yet to be discovered and/or translated to the clinical setting, many commonly-used biomarkers with a definite clinical role in the primary tumor should be assessed for their potential role in their respective metastases for a better stratification of patients with metastatic disease, as well as new studies of validation for biomarkers classically associated with other malignancies than the one subject to study, but potentially harboring clinical value.

Sporadic CRC molecular carcinogenesis is a well-known process to date; a new classification was summarized in 2010 and included three different molecular pathways: a traditional pathway (driven by early *APC* point mutations), an alternative pathway (involving *KRAS* or *APC* mutations), and a serrate pathway (showing early *BRAF* mutations) [99]. On the contrary, no codified pathway has been identified in the metastatic process, but numerous molecular markers have been studied in this setting. Within the subject of liver metastases, a systematic review showed that *KRAS* somatic mutations were a negative prognostic factor for overall and disease-free survival. In addition, twelve studies reported on *BRAF* mutations with a prevalence ranging from 0 to 9.1% in resected liver metastases. *BRAF* hotspot mutations were strongly associated with a worse prognosis. On the contrary, *TP53* and *PIK3CA* gene mutations did not affect outcomes [36]. Recent evidence showed how combined analysis of *KRAS* and *PIK3CA* mutations, MET and PTEN expression in primary tumors and corresponding metastases in colorectal cancer demonstrated discordance between the two sites. In addition, mutations targeting exon 9 of *PIK3CA* in primary tumors and loss of PTEN nuclear expression in metastases are known to be correlated with *KRAS* missense mutations [100]. In metastatic CRC, a recent report speculated that the anti-EGFR treatment could represent a selective pressure which allows the selection of *KRAS*-mutant tumoral subclones. The authors illustrated that tumors which initially respond to anti-EGFR antibodies often develop resistance within several months of therapy [101] and the problem arises whether it is actually a molecular heterogeneity “ab initio” or acquired during treatment. An emerging biological therapeutic agent is represented by panitumumab, a fully human anti-EGFR monoclonal antibody therapeutic designed to treat metastatic colorectal cancer. In this setting, the importance of the evaluation of *KRAS* mutation is capital, given that the use of panitumumab in cases with RAS mutations has been proven to be not beneficial, and possibly harmful. Moreover, the use of this antibody as maintenance therapy and conversion therapy for unresectable liver metastases is gaining consent in the literature [102]. To this end, a multigene, next-generation sequencing-based test has been recently developed and commercialized as a companion diagnostic for panitumumab.

In 2015, an international consortium of experts proposed a gene expression-based classification of CRC, namely the Consensus Molecular Subtypes (CMS) [103]. This classification takes into account different molecular signatures of the primary tumor, such as MSI, hypermutator status, *BRAF* and *KRAS* mutations, WNT and MYC activation, TGF-β activation, and methylation status. Each of the four subtypes (i.e., CMS1, CMS2, CMS3, and CMS4) presents different biological and clinical behavior. In particular, the CMS4 subgroup of patients has been demonstrated to harbor the highest risk of liver metastasis [103,104]. Among these tumors, molecular alterations, which have been observed in CRCLM, are recurrent, such as TGF-β pathway activation trough *SMAD4* genetic alterations. Given the prognostic and predictive significance of this molecular classification, a rational approach for a better stratification of CRCLM patients could be the translation of this categorization, which was developed for the primary tumor, to the metastatic counterpart. This could prove especially beneficial for the 10% of CRC cases that shows private mutations in liver metastases [10]. In this subset of patients, CMS labeling could be different between the primary tumor and the corresponding metastases as a result of the genetic drift [11].

To date, three micro-RNAs (endogenous, small, noncoding, single-stranded, conserved RNA involved in regulation of mRNA targets translation involved in different physiological and tumoral processes including tumor cell growth, invasion, and metastasis by regulating the expression of target genes) [105] have been identified as potential markers for worse prognosis and metastatic dissemination of CRC, with discordant results: miR-200c, miR-196b-5p miR-146a, miR-181a, miR-21, and miR-9. Further correlations with clinical outcomes are needed for a complete validation of the prognostic values of such biomarkers in the setting of CRC liver metastases.

It is also worth noting that a recent study by Qian et al. demonstrated that overexpression of thrombospondin 2 (THBS2), inhibin beta B (INHBB), and biglycan (BGN) led to a worse overall and disease-free survival in metastatic patients, enriching their results with the consideration that chemotaxis, coagulation, and lipid metabolism might play critical roles in the processes of carcinogenesis and liver metastasis [106].

Regarding immunohistochemistry, one of the most promising markers is SFD1 (CXCL12), which apart from its indirect prognostic significance [71], could potentially give a predictive value, given the fact that novel drugs interacting with the CXCL12/CXCR4 axis have been studied in anticancer therapies [107]. Other markers potentially eligible for target therapies include FRalpha (a recent study investigated in vitro and in vivo activity of IMGN853, a drug targeting this biomarker in biologically aggressive endometrial cancers) [108] and NEK2 (inhibition of its kinase activity has been studied in preclinical studies with MBM-5) [109].

Tumoral stromal microenvironment is nowadays considered an important player in cancer tumorigenesis and progression. In this view, alterations of miRNAs in CRC stroma allow forming a permissive background that can permit Tenascin C to promote liver metastasis, identifying it as a novel biomarker to predict postoperative prognosis [98].

Other immunohistochemical markers such as MMP7, Endoglin, Beta-catenin, PPRG, GADD45B, and MET are associated with the development of liver metastases and give only indirect prognostic information, given the fact that patients with the metastatic disease show a worse 5-year survival than nonmetastatic counterparts.

Circulating tumor cells (CTCs) are a subset of tumor cells freely circulating in the peripheral blood; a fraction of them, the metastasis-initiating-cells (MICs), has the capability to form metastasis. The study of MICs phenotype is particularly important for the understanding of the biological mechanism of metastasis and could also potentially harbor prognostic and predictive significance. In a recent study, the CD133^+^ CD44^+^ CD54^+^ subpopulation of CTCs has a prognostic value in metastatic colorectal cancer and shows a significant survival improvement in patients who did not undergo surgical treatment for metastasis [65]. Those studies are particularly promising, considering the feasibility of this test (a simple blood sampling) potentially able to add more prognostic information at subsequent moments during patient monitoring.

Proliferative index assessed with Ki67 has been identified as a prognostic factor in numerous human malignancies, including lung cancer [110]. In colorectal metastatic cancer, Ki67 showed a controversial role in two different studies, which demonstrated its correlation with shorter disease-free survival [77] but no statistically significant association between Ki67 expression and overall survival [75]. Further studies should focus on this well-established prognostic index for other malignancies, given its undoubtedly potential for liver metastases.

The importance of intratumor heterogeneity has been emerging in several studies studying molecular or immunohistochemical expression in a plethora of human malignancies, in particular, lung cancer [110]. In the setting of CRC liver metastases, several studies addressed this topic and demonstrated the importance of intratumor heterogeneity for resistance to treatment [111] and a number of potentially targetable sites [112].

In conclusion, a small number of molecular and immunohistochemical markers have been described in the literature in terms of giving prognostic and predictive information on CRCLM; however, the field appears to be lacking robust biomarkers. For a better stratification of patients with this disease and, potentially, for creating new means of eligibility to the latest treatment advances, further research is needed, both in terms of preclinical (search of novel biomarkers in vitro and in vivo, in model organisms as well as in human tissues) and clinical (validation for prognostic and predictive significance of biomarkers through correlation with patient follow up and response to therapies) studies.

## 4. Materials and Methods

### 4.1. Inclusion Criteria

We focused on recent articles concerning the molecular and immunohistochemical characterization of liver metastases from colorectal carcinoma that demonstrated prognostic and/or predictive implications.

**Figure 5:**
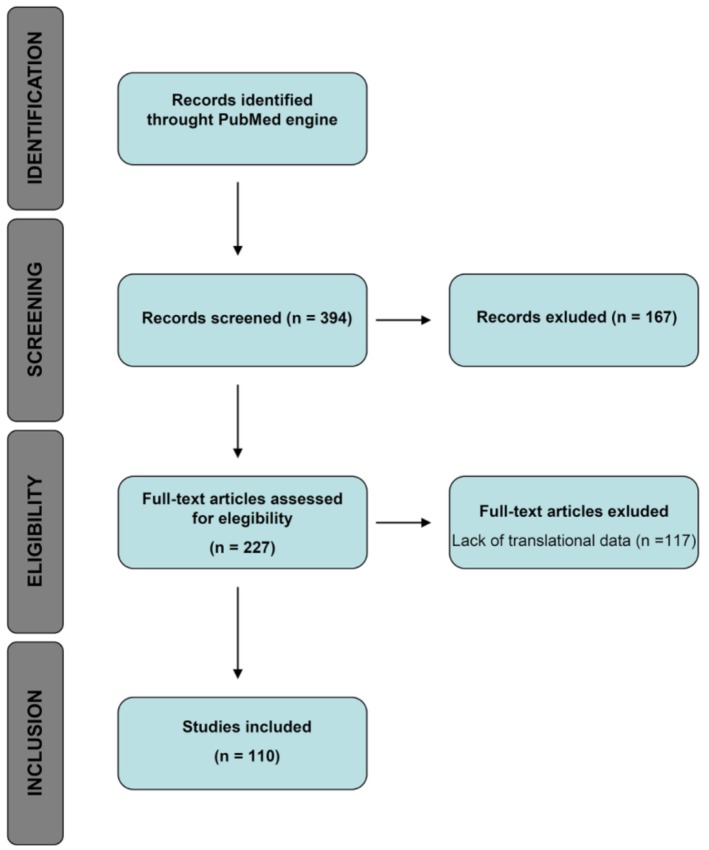
Flowchart of study design.

A flowchart of the study design is illustrated in Figure 5.

### 4.2. Search Terms

Studies were identified using the search engine PubMed (available online: https://www.ncbi.nlm.nih.gov/pubmed). A set of search terms was elaborated in order to identify pertinent studies. Results were also initially filtered by considering only the studies published in English in the last 10 years (2008–2018).

The full search code used was: (((prognostic[All Fields] OR predictive[All Fields]) AND (“biomarkers”[MeSH Terms] OR “biomarkers”[All Fields])) AND (colorectal[All Fields] AND (“adenocarcinoma”[MeSH Terms] OR “adenocarcinoma”[All Fields] OR “cancer”[MeSH Terms] OR “cancer” [All Fields]))) AND ((“liver”[MeSH Terms] OR “liver”[All Fields]) AND (“neoplasm metastasis”[MeSH Terms] OR “neoplasm metastases”[All Fields] OR (“neoplasm”[All Fields] AND (“metastasis”[All Fields] OR “metastases”[All Fields])) OR “neoplasm metastasis”[All Fields] OR “metastases”[All Fields] OR “metastasis”[All Fields])) AND (“2008/07/30”[PDat]: “2018/07/27”[PDat] AND English[lang]).

A total of 394 articles were identified using these criteria.

### 4.3. Exclusion Criteria

A primary screening of the 394 studies was carried out in order to exclude irrelevant articles. Exclusion criteria included: pure morphological studies and studies without prognostic and/or predictive significance. A total of 227 articles endured this initial screening.

Out of the 227 remaining articles, 117 were excluded due to lack of relevant data. In the end, 110 articles were included in this review.
